# Comparative Evaluation of High-Speed Videoendoscopy and Laryngovideostroboscopy for Functional Laryngeal Assessment in Clinical Practice

**DOI:** 10.3390/jcm14051723

**Published:** 2025-03-04

**Authors:** Joanna Hoffman, Magda Barańska, Ewa Niebudek-Bogusz, Wioletta Pietruszewska

**Affiliations:** Department of Otolaryngology, Head and Neck Oncology, Medical University of Lodz, 90-153 Lodz, Polandewa.niebudek-bogusz@umed.lodz.pl (E.N.-B.); wioletta.pietruszewska@umed.lodz.pl (W.P.)

**Keywords:** high-speed videoendoscopy (HSV), laryngovideostroboscopy (LVS), kymographic analysis, phonatory function, vocal fold oscillations, voice disorders, benign glottal lesions, malignant glottal lesions

## Abstract

Advancements in dynamic laryngeal imaging, particularly high-speed videoendoscopy (HSV), have addressed several limitations of laryngovideostroboscopy (LVS). This study aimed to compare the success rates of LVS and HSV in generating recordings suitable for objective functional assessment of vocal fold movements. **Methods:** This study included 200 patients with voice disorders (123 with benign glottal lesions, 56 with malignant lesions, and 21 with functional voice disorders) and 47 normophonic individuals. All participants underwent LVS followed by HSV. Kymographic analysis was performed to evaluate phonatory parameters, including amplitude, symmetry, and glottal dynamics. The success of both methods in generating analyzable kymograms was assessed, and statistical comparisons were made using the chi-square test (significance level set at *p* < 0.05). **Results**: The failure rate for LVS was significantly higher (43.32%) compared to HSV. HSV successfully generated kymograms in 68.22% of cases where LVS failed. The primary factors contributing to LVS failure included synchronization issues, inadequate recording brightness, unstable phonation, and hidden glottal opening. Failure rates related to structural obstacles were similar between the two methods. HSV demonstrated superior kymogram feasibility across all subgroups, with the highest success observed in cases of organic glottal pathologies (30.73%). A significant advantage of HSV was observed for both benign and malignant glottal lesions, especially in cases of asynchronous vocal fold oscillations. **Conclusions**. By overcoming the inherent limitations of LVS, HSV provides a more reliable and objective assessment of phonatory function. Its ability to generate suitable kymograms with greater precision makes HSV a valuable tool for routine clinical diagnostics, enabling the accurate identification of subtle laryngeal pathologies and enhancing diagnostic accuracy.

## 1. Introduction

The assessment of voice function necessitates a comprehensive array of diagnostic tools, encompassing perceptual voice evaluation, subjective patient assessments, acoustic measurements, and laryngostroboscopic examination [1]. Each modality provides unique insights into vocal function, reflecting the complexity of voice diagnostics. Among these, the accurate visualization of vocal fold vibrations remains pivotal for diagnosing and managing laryngeal disorders [1,2,3]. Of the available visualization techniques, laryngovideostroboscopy (LVS) and high-speed videoendoscopy (HSV) are particularly valuable for evaluating the dynamic function of the glottis.

Laryngovideostroboscopy is the standard imaging technique for assessing phonatory movements of the vocal folds and remains widely used in clinical practice [1]. This method relies on digital video recordings that reconstruct vocal fold movement over multiple phonation cycles. However, LVS does not provide real-time visualization of vocal fold function. Instead, it generates an averaged representation of the phonation cycle by combining sequential frames from multiple cycles, capturing minor variations that may occur. As such, LVS is most effective when vocal fold vibrations are regular and synchronous and when stable phonation samples of sufficient duration can be recorded [4,5].

A stroboscopic visual-perceptual assessment via LVS is recommended for analyzing various aspects of vocal fold function, including vibratory amplitude, mucosal wave propagation, phase symmetry, vertical positioning, and glottal closure patterns. However, as noted by the European Laryngological Society, the interpretation of LVS findings can be affected by observer bias, potentially compromising objectivity [1]. The use of blinded evaluations or panel-based ratings, particularly in post-surgical follow-ups, may improve their reliability, though such practices are challenging to implement in routine clinical settings. The American Speech–Language–Hearing Association also underscores the importance of integrating quantitative measures into vocal fold assessments to enhance diagnostic precision as technological advancements progress [6].

High-speed videoendoscopy (HSV) addresses many limitations inherent in LVS by providing real-time imaging of vocal fold vibrations. This technology captures detailed and reliable data on vocal fold dynamics, enabling the assessment of both synchronous and asynchronous phonatory patterns [7,8,9]. HSV operates at frame rates higher than 2000 frames per second, offering high-resolution visualization of vocal fold oscillations. Unlike LVS, which captures approximately 10 phonatory cycles over a 10 s interval, HSV achieves comparable detail within just one-tenth of a second [5,10,11,12,13].

This capability allows HSV to analyze vocal fold function even in challenging cases, such as dysphonic voices, irregular glottal movements, or short phonation times, which are often inadequately captured by LVS. Furthermore, HSV recordings generate high-quality kinematic images that enable more accurate kymograms—graphical representations of vocal fold movement over time and space [14,15,16,17,18,19]. Kymography derived from HSV is widely regarded as one of the most effective methods for objectively assessing the temporal aspects of phonatory function [10].

While kymographic analysis can also be applied to LVS recordings (termed strobovideokymography), this process is labor-intensive and depends on the availability of high-quality stroboscopic images [4,5]. HSV offers a significant advantage in this regard due to its ability to produce kymographic data from brief recording sessions, thereby expanding the range of patients who can undergo this type of analysis [11].

This study aims to compare the success rates of obtaining LVS and HSV recordings suitable for objective evaluation in routine clinical practice. The analysis focuses on identifying the causes of examination failure across the entire study cohort, stratified by patient categories including normophonic individuals, as well as those with functional, benign, and malignant vocal fold lesions.

## 2. Materials and Methods

### 2.1. Study Group

This study included 247 patients from the Department of Otolaryngology, Head and Neck Oncology at the Medical University of Lodz and its affiliated outpatient clinic, evaluated from 2020 to 2023 for various laryngeal lesions. The mean age of the total group was 58.6 years (median 62, range 21–90). A control group was established, comprising 47 normophonic subjects with no history of dysphonia and no organic or functional abnormalities of the larynx. The study group consisted of 200 patients with voice disorders, including 123 with benign glottal lesions, 56 with malignant lesions, and 21 with functional voice disorders. All patients with organic lesions underwent laser microsurgery, confirming the initial diagnosis histopathologically. The inclusion criteria for the study group encompassed the following: age ≥ 18 years, the ability to perform HSV and LVS tests without structural barriers (e.g., hypopharyngeal or epiglottic tumors obstructing the glottis), and patient cooperation. After each examination, attempts were made to generate kymograms. Detailed group characteristics are shown in Table 1.

This study received approval from the Ethical Committee of the Medical University of Lodz (no. RNN/96/20/KE, dated 8 April 2020), and written informed consent was obtained from all participants.

### 2.2. Methods of Examination

All patients first underwent an otolaryngological examination, including a comprehensive interview regarding voice-related issues. Each subject then underwent a baseline endoscopic examination of the larynx under white light, followed by a strobe light examination (LVS) using the same endoscope. Laryngovideostroboscopy was performed using a rigid 90° endoscope (Olympus WA96105A, Olympus Medical Systems, Tokyo, Japan). In select cases where rigid endoscopy was not feasible due to anatomical constraints or patient discomfort, a flexible endoscope (Olympus ENF-VH2, Olympus Medical Systems, Tokyo, Japan) was used. Digital imaging was recorded with an Olympus Visera Elite OTV-S190 camera (Olympus Medical Systems, Tokyo, Japan) paired with a xenon light source and an Olympus CLL-S1 (Olympus Medical Systems, Tokyo, Japan) strobe lamp. Following LVS, subjects were examined with an HSV camera. HSV images were recorded using the Advanced Larynx Imager System (ALIS) [20] equipped with laser diode lighting (ALIS Lum-MF1, Diagnova Technologies, Wroclaw, Poland) and a high-speed camera (ALIS Cam HS-1, Diagnova Technologies, Wroclaw, Poland) connected to a rigid oval endoscope (Fiegert–Endotech ϕ12.4/7.2, Tuttlingen, Germany) with a light guide using a 4.8 mm fiber optic cable.

High-speed videoendoscopy (HSV) was performed using a color high-speed camera with a frame rate of 4000 frames per second (fps) and a pixel resolution of 512 × 512. This configuration allowed for high temporal and spatial resolution, facilitating the precise analysis of vocal fold vibrations. For laryngovideostroboscopy (LVS), images were captured at a rate of one stroboscopic cycle per second, ensuring that periodic vocal fold vibrations were effectively reconstructed, with a pixel resolution of 480 × 400. The stroboscopic system used an LED light source synchronized with the patient’s fundamental frequency to achieve optimal imaging conditions. Regarding the duration of analyzed sequences, LVS recordings required a minimum phonation time of 10 s to ensure the capture of sufficient vibration cycles for meaningful analysis. In contrast, HSV recordings were completed within 0.06–0.08 s, corresponding to 10–20 phonation cycles (depending on the fundamental frequency), thereby enabling the real-time visualization of vocal fold oscillations in a much shorter time frame.

**Figure 1:**
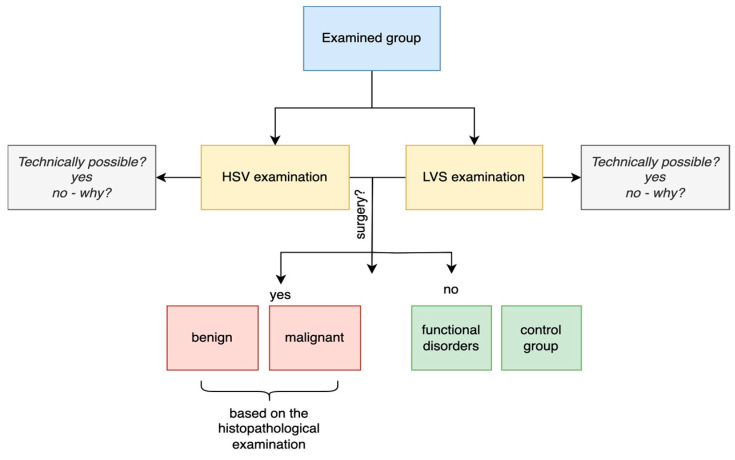
Sequential steps of the study protocol.

Among the 200 subjects with dysphonia, 178 with hypertrophic glottal pathologies underwent laser microsurgery, and final diagnoses were based on histopathological examination of tissue specimens. The classification of benign lesions adhered to the WHO 2017 dysplasia grading, while all malignant lesions were confirmed as squamous cell carcinoma. The study design was applied to both the control and study groups (Figure 1).

Figure 1 illustrates the sequential steps of the study protocol, outlining the diagnostic workflow applied to all participants. The process commenced with an initial otolaryngological examination, including a comprehensive medical interview to assess voice-related symptoms. Following the baseline assessment, each participant underwent laryngovideostroboscopy (LVS) as the first imaging modality, which was subsequently followed by high-speed videoendoscopy (HSV).

The examination sequence was standardized for all subjects to ensure consistency and minimize potential biases. The LVS examination involved recording vocal fold vibrations using strobe light synchronization, whereas the HSV examination captured high-frame-rate recordings to assess real-time vocal fold dynamics. After data acquisition, kymographic analysis was performed on both LVS and HSV recordings to evaluate phonatory parameters, such as vibratory amplitude, symmetry, and glottal closure.

Additionally, the protocol included specific criteria for determining successful kymogram generation, focusing on the quality of the extracted images and their suitability for objective assessment. The study protocol also accounted for potential challenges, such as insufficient phonation stability or anatomical obstructions, which were systematically documented and analyzed.

#### 2.2.1. Differences Between LVS and HSV Recordings

**Figure 2:**
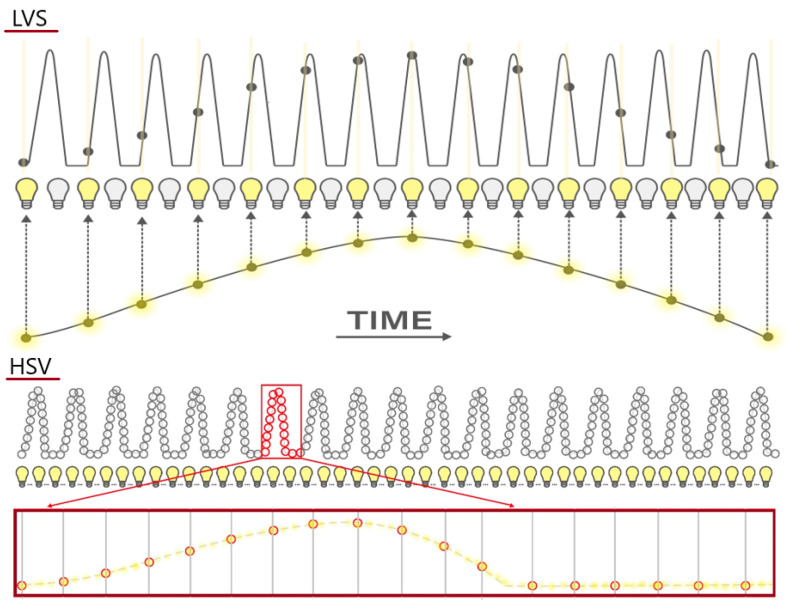
Schematic illustration of the creation of a videostroboscopic and high-speed video recording, adapted and modified based on the illustration from Deliyski (2010) [16].

LVS synchronizes strobe light with the patient’s voice frequency to produce an apparent slow-motion image of vocal fold vibrations. However, this is achieved by capturing frames from different phonation cycles, often from widely separated points in the cycle. (Figure 2—adapted and modified based on the illustration from Deliyski (2010) to provide a clearer representation of HSV functionality within the context of our study. The original source has been accordingly cited to acknowledge the contribution [16].) As a result, LVS imaging requires regular vibration periodicity to produce accurate results. This dependence makes LVS unsuitable for assessing irregular vocal fold vibrations or short, stable phonation cycles in patients with severe dysphonia, where the glottal image may appear blurred, resembling static light endoscopy. Additionally, the recorded sample must be long enough to yield a reliable image. These limitations were observed in patients where LVS could not be conducted due to unstable phonation. Similar challenges are noted in the literature [21].

**Figure 3:**
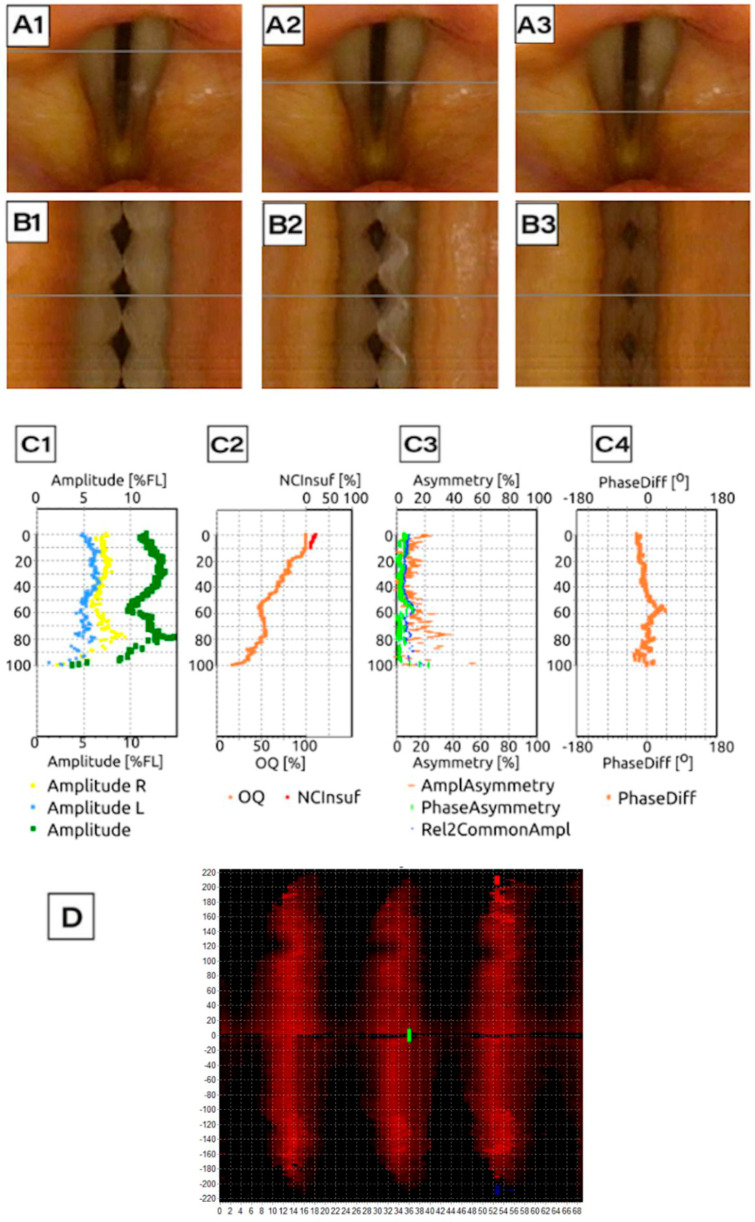
Analysis of data generated on the basis of an LVS record for Subject 1 (normophonic patient, 40 years old woman with 3 cycles of vocal folds movement); (**A1**–**A3**) image of the glottis with the reference line along the course of the vocal folds marked with a gray line, for which a videokymogram was obtained at the posterior, middle, and anterior sections, respectively; (**B1**–**B3**) videokymograms generated at the marked sections: the posterior, middle, and anterior parts of the glottis, respectively; (**C1**) graphs showing (*y*-axis presents the glottal axis) the amplitude movement of the vocal folds: blue—right vocal fold, yellow—left vocal fold, and green resultant of the movement of both vocal folds; (**C2**) graph showing the degree of glottal closure: orange line—value of Open Quotient alongside the longitudinal axis of the glottis and red line—non-Closure Quotient determining the insufficiency of the glottis; (**C3**) asymmetry rate; (**C4**) phase difference; (**D**) phonovibrogram: a diagram presenting the movement of the vocal folds in time in relation to the center of the glottis; the center of the diagram indicates the anterior part of the glottis; the upper part describes the movement of the left vocal fold, while the lower part describes the movement of the right vocal fold.

High-speed videoendoscopy (HSV) overcomes these limitations by recording the actual vibrations of the vocal folds at a speed of several thousand frames per second, enabling the detailed visualization of successive phonation cycles. HSV thus facilitates the analysis of vibratory cyclicity over extended periods in asynchronous voices, where strobe light synchronization is ineffective. This method is applicable for both irregular glottal function and severe vocal fold pathology, where patients may not sustain a stable phonation for long periods. While LVS typically requires capturing 10 cycles over a 10 s period for a fundamental frequency of 100 Hz, the duration may vary depending on the strobe unit settings, which can range from 0.5 to 1.5 cycles per second. In contrast, HSV records the same data within a fraction of a second, independent of cycle synchronization settings. These recordings provide a robust basis for generating a kymographic analysis of successive phonatory cycles (Figure 3).

#### 2.2.2. Kymographic Analysis

**Figure 4:**
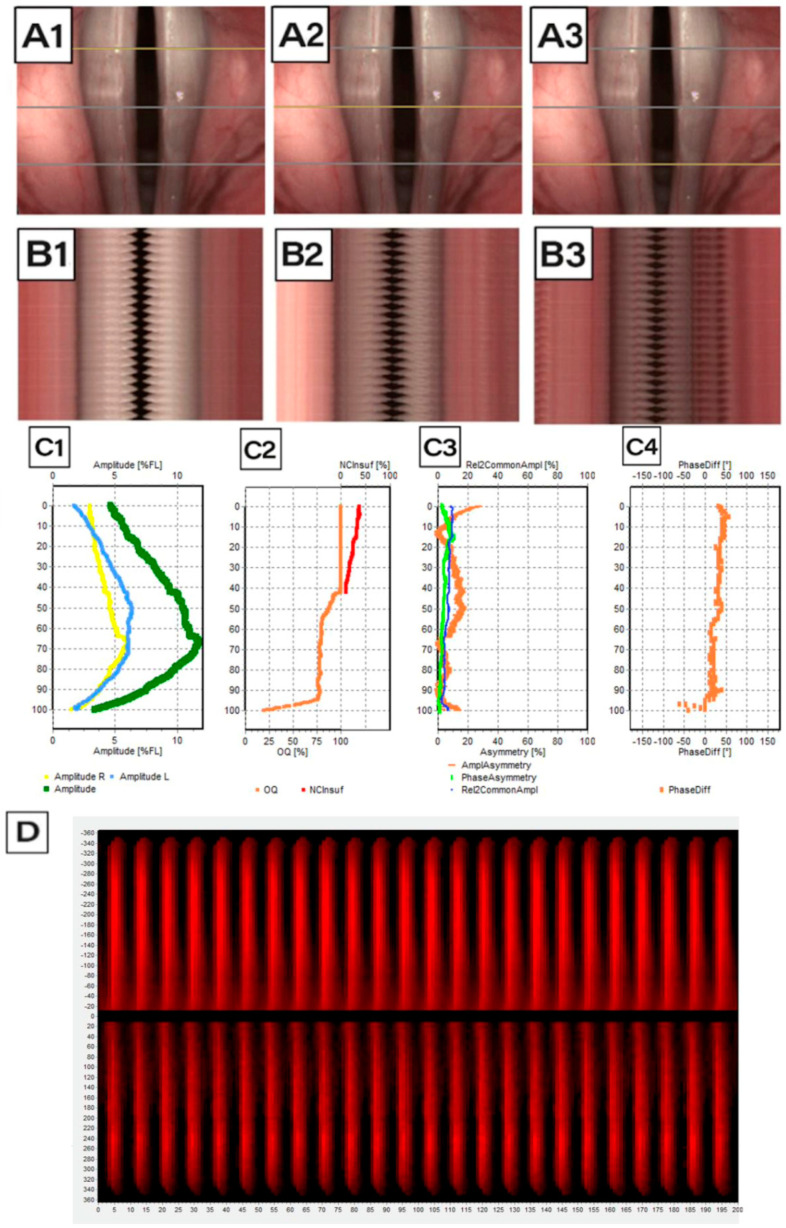
Analysis of data generated on the basis of an HSV record for Subject 1 (normophonic patient, 40 years old woman; 21 cycles of vocal folds movement): (**A1**–**A3**) image of the glottis with the cross-section along the course of the vocal folds marked with a gray line, for which a videokymogram was obtained at the posterior, middle, and anterior sections, respectively; (**B1**–**B3**) videokymograms generated at the marked sections: the posterior, middle, and anterior parts of the glottis, respectively; (**C1**) graphs showing (*y*-axis presents the glottal axis) the amplitude movement of the vocal folds: blue—right vocal fold, yellow—left vocal fold, and green resultant of the movement of both vocal folds; (**C2**) graph showing the degree of glottal closure: orange line—value of Open Quotient alongside the longitudinal axis of the glottis and red line—non-Closure Quotient determining the insufficiency of the glottis; (**C3**) asymmetry rate; (**C4**) phase difference; (**D**) phonovibrogram: a diagram presenting the movement of the vocal folds in time in relation to the center of the glottis; the center of the diagram indicates the anterior part of the glottis; the upper part describes the movement of the left vocal fold, while the lower part describes the movement of the right vocal fold.

Following image acquisition, LVS and HSV recordings were subjected to detailed kymographic analysis using DiagNova Technologies software version 1.3 [20]. Kymographic section plots were generated at three levels along the entire vertical axis of the vocal folds (posterior, middle, and anterior) for both LVS and HSV recordings (Figure 3(A1–A3) and Figure 4(A1–A3)). A sample analysis for a normophonic subject based on LVS is shown in Figure 3.

Each LVS sample, after semi-automatic image stabilization, required manual preparation by an ENT specialist or phoniatrician. This process involved outlining the vocal fold edges and glottal area and adjusting individual frames as necessary. Following this, the software generated a kymographic cross-section at 1/3, 2/3, and 3/3 of the glottal length (Figure 3(B1–B3)), which was subsequently digitally equalized for brightness due to fluctuations in strobe light intensity. At least three full recording cycles were necessary to generate an LVS-based kymographic analysis, but often, more cycles were required. This limitation affects the reliability of the results from strobovideokymography.

The usability of a kymogram was determined based on the clear visualization of vocal fold oscillations without artifacts that could impair quantitative analysis. A kymogram was considered usable if it allowed for the extraction of phonatory parameters, such as amplitude, symmetry, and glottal closure, with sufficient clarity and accuracy to support objective functional assessment. In contrast, unsuccessful kymogram generation was defined as cases where visualization was obscured due to factors such as excessive movement artifacts, hidden glottal opening, or synchronization issues that prevented accurate analysis.

Kymograms derived from HSV recordings are simpler and faster to produce. Unlike LVS, which requires multiple cycles, HSV allows for kymogram generation even with short phonation times. Additionally, HSV kymograms directly correspond to the selected phonation cycles, providing more precise data on actual vocal fold movements at specific glottal levels during each cycle phase (Figure 4(B1–B3)). Due to the shorter recording time required for HSV (0.06–0.08 s of phonation), glottal axis correction was not necessary. The stabilized view in HSV recordings ensured a stable position of the glottis, reducing the risk of human error and enhancing kymogram quality and accuracy.

Phonovibrograms, which graphically represent vocal fold movement, were also generated based on both LVS and HSV recordings (Figure 3D and Figure 4D). Phonovibrograms effectively consolidate data on vocal fold oscillations in a color-coded map over a specified time interval [22]. The *X*-axis represents time, while the *Y*-axis represents points along the vocal fold edge. In the map, red intensity indicates the degree of vocal fold opening (the momentary distance between the vocal fold edge and the glottis axis), while black denotes that the distance equals zero and the vocal fold is aligned to the glottal axis center. A comparison of LVS-based and HSV-based phonovibrograms shows that HSV provides a clearer, more detailed visualization of vocal fold oscillations.

#### 2.2.3. Statistical Analysis

A comprehensive statistical analysis was performed to evaluate the effectiveness of LVS and HSV in generating analyzable kymograms. Descriptive statistics were calculated for all measured parameters, and categorical variables were compared using the chi-square test to determine statistical significance. A *p*-value of <0.05 was considered statistically significant. All statistical analyses were conducted using GraphPad Prism software version 9.3.1. The statistical methodology ensured robust comparisons between the two imaging modalities, enabling the accurate assessment of their diagnostic capabilities.

## 3. Results

**Figure 5:**
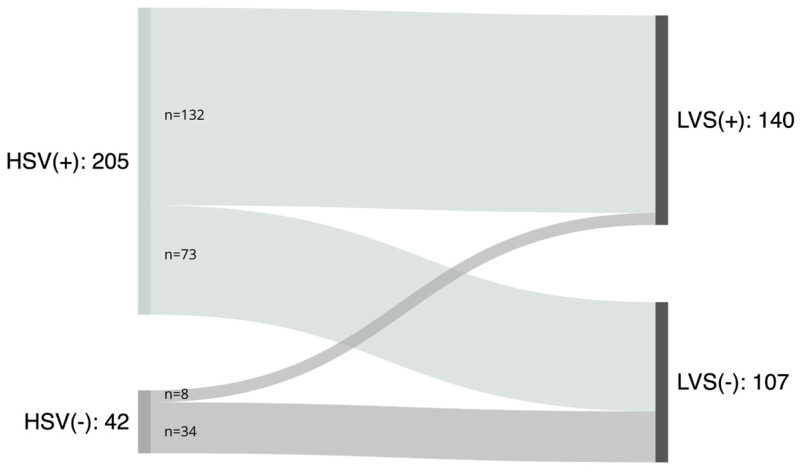
Groups of patients qualified for the study: HSV(+)—successful HSV examination; HSV(−) unsuccessful HSV examination; LVS(+)—successful LVS examination; LVS(−)—unsuccessful LVS examination.

In this study, it was possible to generate kymographic records for 205 patients (82.99% of the total) using HSV, compared to 140 patients (56.68%) using LVS (Figure 5), and a statistically significant difference favoring HSV (*p* < 0.001) was found.

Only eight patients (3.24%) had kymograms generated solely using LVS without concomitant HSV results. In all these cases, anatomical or structural obstacles, such as a prominent epiglottal pedicle or a flat epiglottis obscuring the anterior vocal folds, impeded HSV imaging due to the limitations of rigid optics. This challenge was mitigated in LVS by using flexible optics.

**Figure 6:**
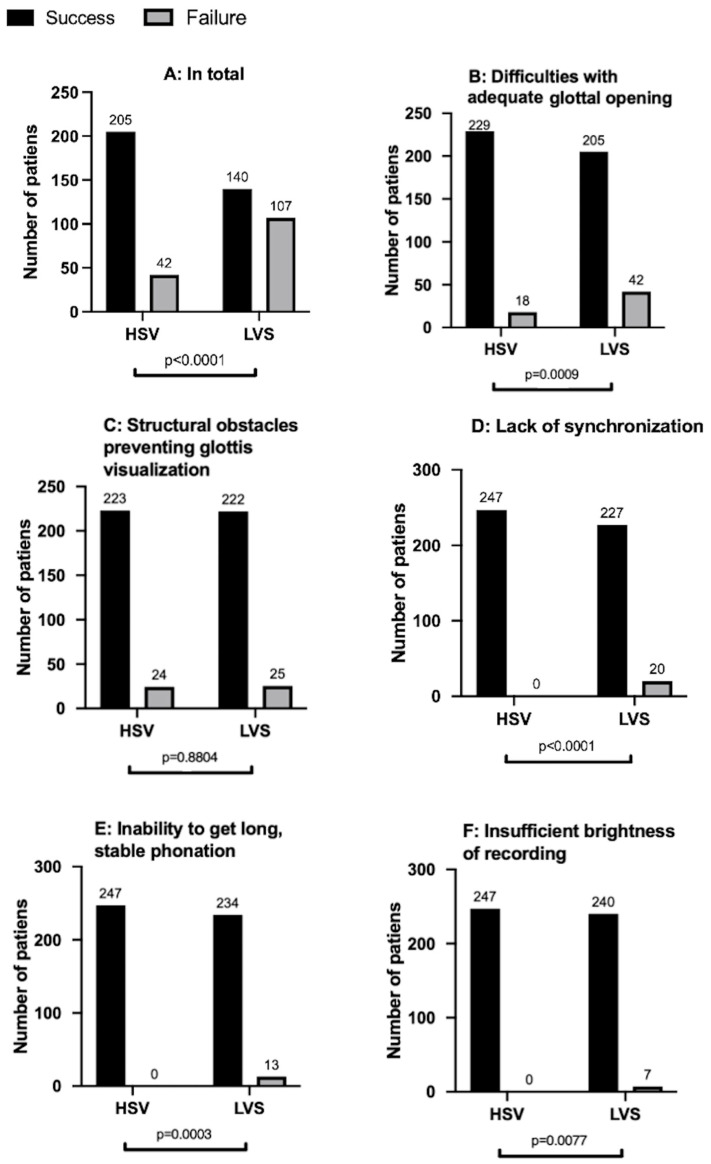
Difficulties with kymographic analysis in the HSV and LVS groups (total group n = 247): (**A**) for the whole study group and (**B**–**F**) for the following difficulties described in Section 3.1.

**Figure 7:**
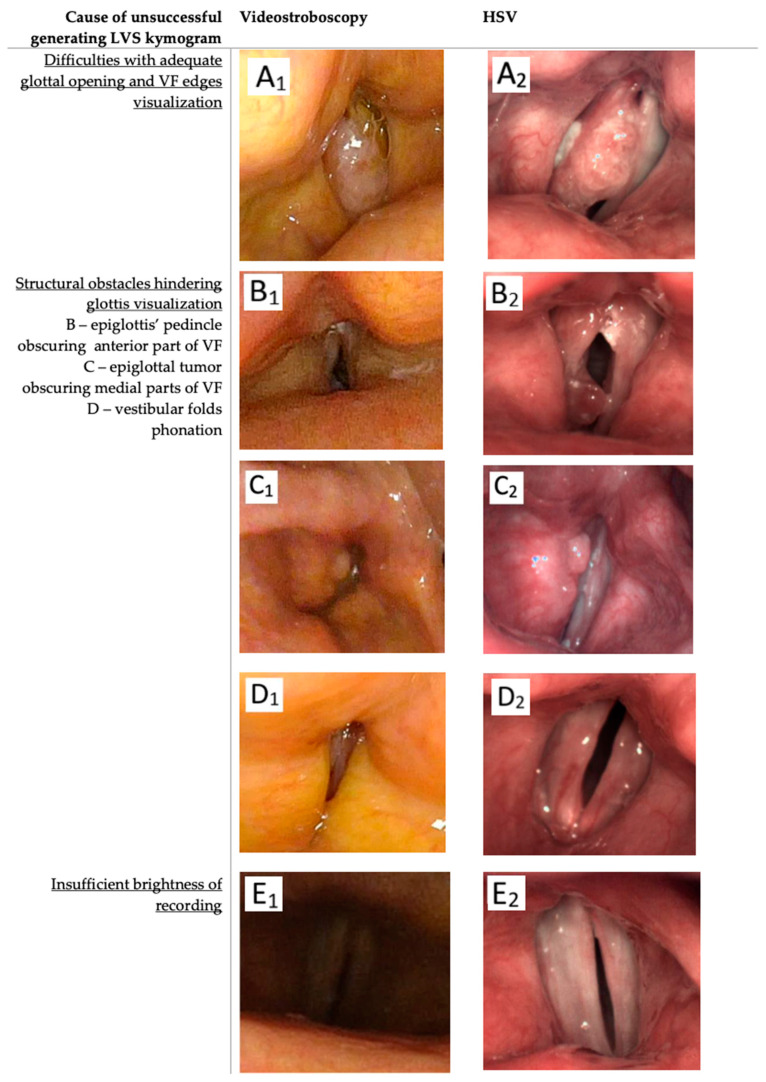
Images of patients’ larynx in whom generating LVS-based kymogram was insufficient to generate kymograms (**A1**,**B1**,**C1**,**D1**,**E1**) compared with images from successful HSV-based kymography (**A2**,**B2**,**C2**,**D2**,**E2**). Subject (**A**): a male patient with carcinoma planoepitheliale G-3 in the right vocal fold; Subject (**B**): a male patient with a fibrovascular polyp in the right vocal fold; Subject (**C**): a male patient with epiglottal carcinoma planoepitheliale G-2; Subject (**D**): a female patient with functional voice disorders; Subject (**E**): a male patient with vocal fold inflammation and incomplete glottal closure, resulting in too short stable phonation to obtain an LVS recording. The causes of unsuccessful LVS-based kymogram creation are described in the table. VF—vocal folds.

However, both LVS and HSV examinations failed to produce kymograms suitable for analysis in 34 patients (13.77%). We analyze and discuss the reasons for the difficulties encountered with HSV and LVS testing (Figure 6 and Figure 7).

### 3.1. Observed Issues

#### 3.1.1. Challenges with Adequate Glottal Opening and Vocal Fold Visualization (Figure 7A)

Generating a kymogram requires a clear visualization of the glottal area to define the medial edges of the vocal folds. Hidden glottal opening was observed in both LVS and HSV recordings. In the LVS group, this limitation prevented kymogram generation in 42 patients (42/205; 20.48%), whereas it impacted only 18 patients in the HSV group (18/229; 8.61%), and a statistically significant difference (*p* < 0.001) was found. This issue was especially prevalent among patients with large lesions. Additionally, visualizing the entire vocal fold structure, including the lower edge, was challenging in some cases. While both LVS and HSV theoretically provide similar laryngeal views, clinical experience and technical factors suggest that the HSV system more frequently allows for clear visualization of the lower vocal fold edge. This advantage is likely due to HSV’s ability to capture phonation at lower fundamental frequencies with higher temporal and spatial resolution, reducing the impact of vibratory irregularities that may blur the stroboscopic image.

#### 3.1.2. Structural Obstacles Hindering Glottis Visualization (Figure 7B–D)

Structural abnormalities or variations in the supraglottal region, such as a pedunculated epiglottis obstructing the anterior glottal part, a flat epiglottis, or intrinsic pathologies like vocal fold tumors, impeded glottal visualization. This issue divided patients into two subgroups: The first included those with anatomical variations like an omega-shaped epiglottis or an epiglottal pedicle covering the anterior commissure, obstructing the glottis (Figure 7(B1,B2). The second subgroup (Figure 7(C1,C2)) included patients whose glottal visualization was hindered by extensive pathologies like large glottal tumors (Figure 7(D1,D2)).

Vestibular fold phonation was more common in LVS recordings (Figure 7(D1)), observed even in cases where flexible optics were used for improved visualization, whereas HSV was more affected by rigid optics, particularly in cases of a drooping or omega-shaped epiglottis. Furthermore, extensive proliferative lesions, such as carcinomas that obscured both vocal folds, limited the use of both LVS and HSV in clinical practice due to the inability to generate reliable kymograms for these cases.

#### 3.1.3. Lack of Light Synchronization (Figure 6D)

The synchronization of strobe light with the patient’s vocal frequency is essential for LVS. In 20 patients (8.1% of the total), this was the primary reason for failed LVS kymograms. However, this issue was not present in HSV recordings visualizing the actual motion of the vocal folds, thus eliminating the need for light synchronization.

#### 3.1.4. Inability to Sustain Long, Stable Phonation (Figure 6E)

This issue affected only LVS recordings. Generating high-quality LVS images requires at least 10 s of stable phonation, which some patients with extensive lesions could not maintain. This hindered the ability to assess mucosal wave movement and identify asymmetries. In the LVS group, this problem impacted 13 patients (5.26%).

In Figure 6, “long phonation” is defined as the ability of the patient to sustain a stable phonatory sound for at least 10 s. The “Lack of synchronization” refers to the inability of the strobe light to properly align with the vocal fold vibration frequency, leading to inconsistent or blurred images. These factors were rated by two experienced otolaryngologists and phoniatricians (EN-B and WP) based on the criteria of the instrumental assessment of the voice, ensuring consistency and reliability in the assessment [6].

#### 3.1.5. Insufficient Recording Brightness (Figure 6F)

This issue was not encountered in HSV recordings due to the laser illumination for sufficient laryngeal brightness [23]. However, in the LVS group, insufficient brightness affected seven patients (2.83%).

#### 3.1.6. Summary of Observed Issues

To summarize the evaluation of the unsuccessful generation of LVS kymograms in comparison to HSV-based kymograms (Figure 6), the total failure rate was significantly higher (*p* < 0.001) and resulted in a failure rate of 43.32% (failure of LVS recordings—107/247). The significant difference was observed for the following reasons: hidden glottal opening: *p* = 0.009 (LVS 42/247 vs. HSV 18/247), lack of synchronization: *p* < 0.001 (20/247 vs. 0/247), insufficiently long phonation: *p* = 0.0003 (13/247 vs. 0/247), and insufficient brightness of recordings: *p* = 0.0077 (7/247 vs. 0/247). No significant differences were found for structural obstacles obscuring the glottis *p* = 0.8804 (25/247 vs. 24/247). In our analysis, we categorized visualization challenges into two distinct groups:

1. Hidden glottal opening: This category includes cases where the vocal folds failed to sufficiently open to allow for effective kymographic analysis. This issue primarily arose in patients with functional dysphonia or glottic pathologies that restricted the natural opening of the vocal folds during phonation;
2. Structural Hindrances in Glottal Visualization: this category encompasses cases where visualization was obstructed by anatomical structures such as an omega-shaped epiglottis, a large epiglottic petiole, or supraglottic hyperfunction, leading to incomplete or distorted views of the glottis.

For HSV, the main obstacles were structural hindrances in glottal visualization (24/223; 9.72%) and hidden glottal opening (18/229; 7.28%). HSV exhibited a success rate of 83.00%, a 26.31% higher success rate than LVS. Notably, HSV significantly outperformed LVS in most of the observed challenges (Figure 6).

### 3.2. Analysis of Cases with Inconclusive Kymograms Using Only One Technique

In cases where initial kymogram generation was unsuccessful with both HSV and LVS, we further explored whether optimized HSV and LVS settings and improved patient cooperation could allow for successful kymogram acquisition, especially in patients where one method had already proven inadequate. Among the 107 patients for whom LVS kymograms were not feasible, reliable kymograms were obtained in 73 patients (68.22%) through HSV. However, in 34 cases, neither HSV nor LVS produced a kymogram.

In eight cases where HSV kymograms could not be generated, LVS was successful. In six of these cases, structural obstacles, such as a prominent epiglottis, obstructed HSV visualization, and in the remaining two, hyperfunctional phonation enabled sufficient opening of the glottis.

### 3.3. Comparison of Kymogram Feasibility by Lesion Type

**Figure 8:**
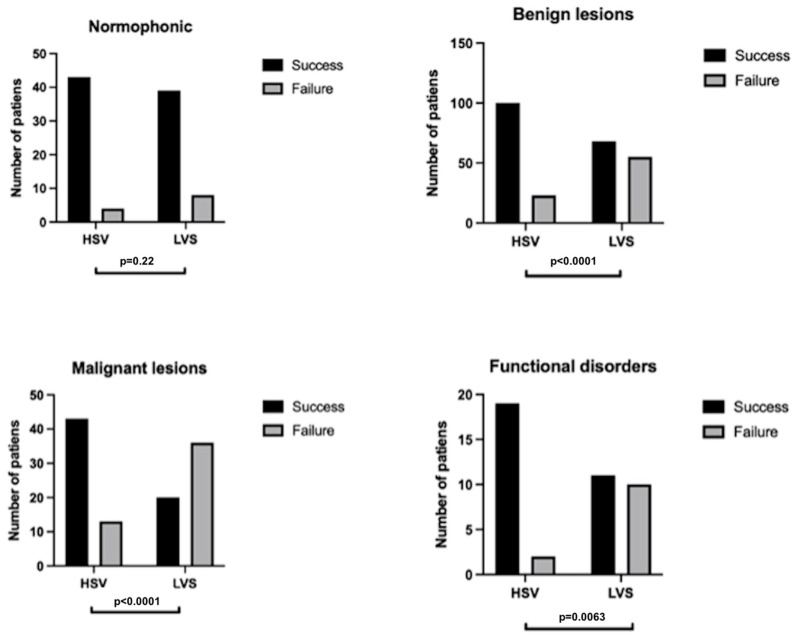
Feasibility of obtaining the kymograms from LVS recordings in comparison to HSV in the patients’ groups divided by diagnosis.

HSV demonstrated a notable advantage in generating kymograms, particularly in patients with organic glottal pathologies. In normophonic patients, HSV yielded additional (in comparison to LSV) kymograms in four out of forty-seven patients (8.51%), while in those with benign lesions, 32 additional kymograms were obtained (26.02%). For patients with malignant lesions, HSV generated additional kymograms in 23 cases (41.07%). A significant difference (*p* < 0.0001) was observed for both benign and malignant hypertrophic masses of the glottis. For patients with functional voice disorders, HSV generated six additional kymograms (28.57%) compared to LVS (*p* = 0.006). The success rate difference across all examined groups is presented in Figure 8.

In all patient groups with organic lesions, the HSV method resulted in a 30.73% higher rate of kymogram generation. Notably, failure causes such as insufficient light, synchronization issues, and insufficient phonation cycles did not occur in HSV recordings.

**Figure 9:**
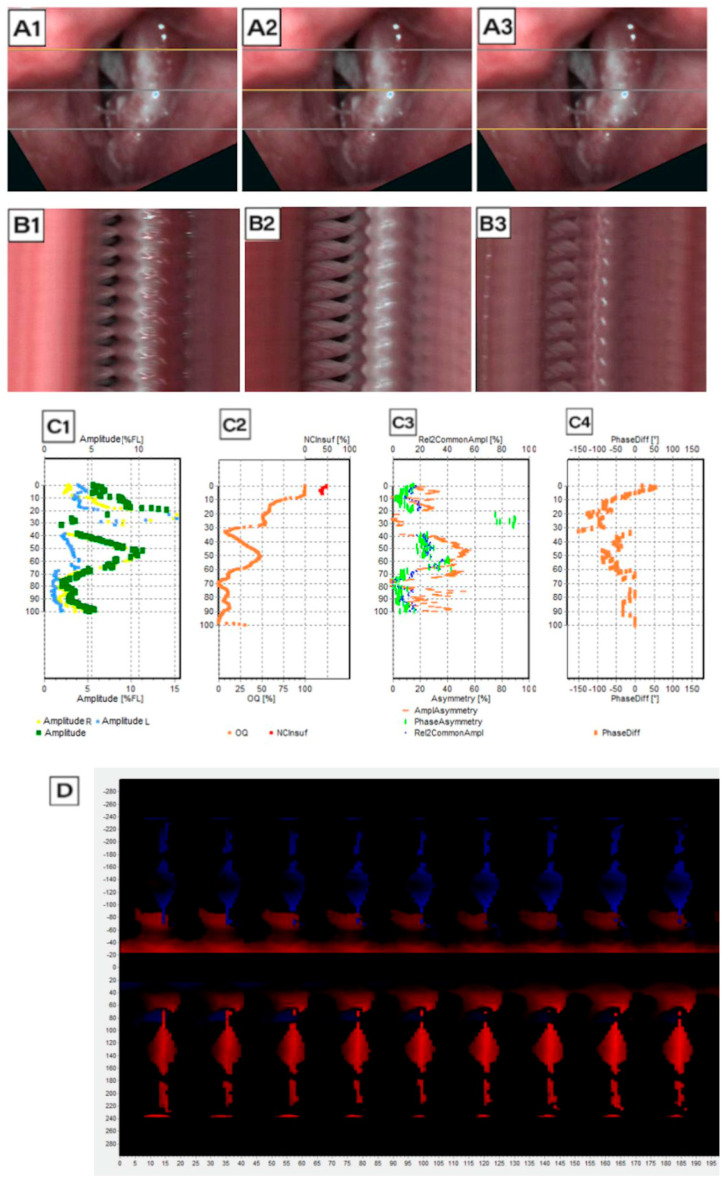
Analysis of data generated on the basis of HSV record for Subject 3: Patient with malignant lesion (61 years old man with cancer of the left vocal fold). Representative kymographic analysis derived from HSV recordings with 9 cycles of vocal fold movement. For a detailed description of the analysis process, please refer to the caption of Figure 3 and Figure 4.

**Figure 10:**
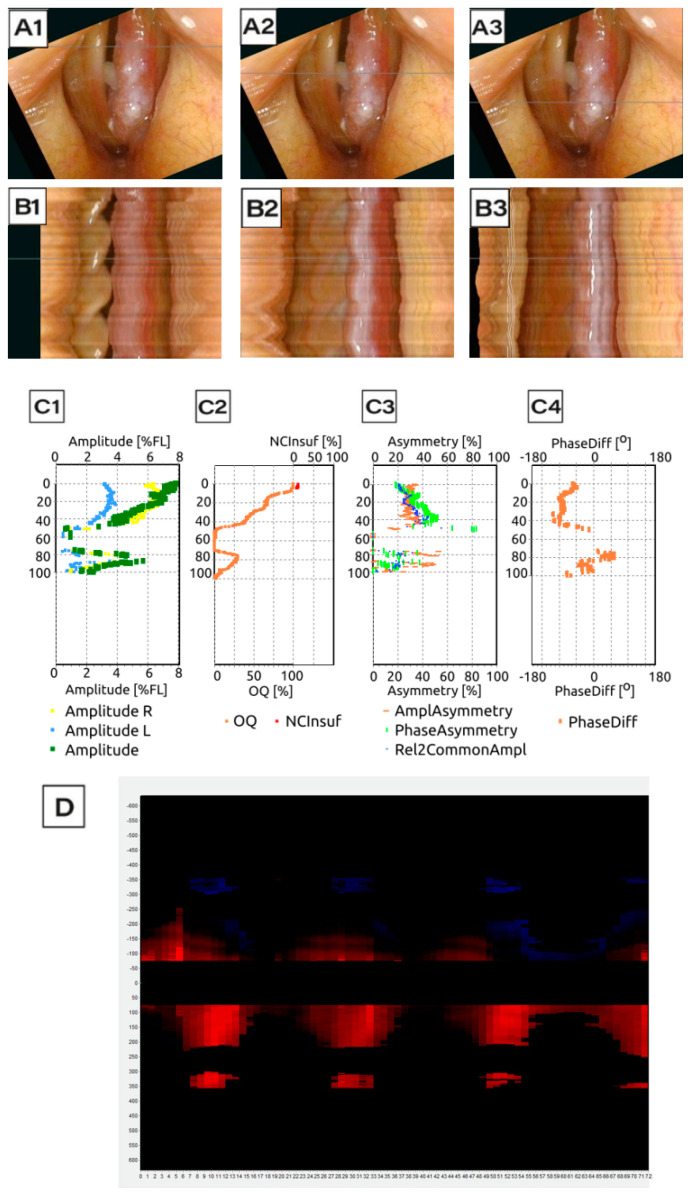
Analysis of data generated on the basis of HSV record for Subject 3: Patient with malignant lesion (61 years old man with cancer of the left vocal fold). Representative kymographic analysis derived from LVS recordings with 3 cycles of vocal fold movement. For a detailed description of the analysis process, please refer to the caption of Figure 3 and Figure 4.

Among patients with carcinoma, hidden glottal opening was a common cause of failure in LVS compared to HSV (Figure 7(A1,A2)), with a difference in occurrence of 29% in carcinoma cases. Moreover, kymograms generated by HSV (Figure 9) provided superior temporal resolution, enhanced visualization of asynchronous vibrations, and greater structural detail compared to LVS kymograms (Figure 10), which rely on periodic phonation cycles and may obscure irregularities. The parameters presented in Figure 3, Figure 4, Figure 9 and Figure 10 were computed using a dedicated kymographic analysis software, which applies semi-automated edge detection algorithms to track vocal fold movement along pre-defined glottal axes. The amplitude, symmetry, and phase differences were calculated based on pixel intensity changes over time, allowing for an objective assessment of vocal fold vibrations. Each computed value represents an average over multiple phonatory cycles to improve measurement accuracy.

The phonovibrograms displayed in these figures provide a comprehensive visualization of vocal fold oscillation patterns over time. They are two-dimensional spatiotemporal representations of changes in the glottal area during phonation; the horizontal axis is time, and the vertical axis represents the length along the glottis. They represent the numeric value of the width of the space between the vocal folds (glottal gap). Darker color indicates decreased amplitude of vocal fold oscillations and increased glottal gap. They consolidate multiple vocal fold oscillation parameters into a single graphical representation, highlighting temporal variations in amplitude and phase relationships. This enables clinicians to visualize asymmetries, irregular vibrations, and insufficient glottal closure.

The last phase of our analysis compared the feasibility of generating kymograms from LVS and HSV recordings in patients with unilateral and bilateral lesions. The data showed a significant difference in kymogram generation success rates between HSV and LVS for both lesion types. Specifically, HSV achieved a 30% higher success rate than LVS, with HSV generating 115 successful recordings compared to 72 for LVS in unilateral lesions and 26 compared to 14 for bilateral lesions. These differences were statistically significant: *p* < 0.00001 for the unilateral lesions and *p* = 0.0058 for the bilateral lesions.

## 4. Discussion

High-speed videolaryngoscopy has become a valuable tool for visualizing vocal fold vibrations, providing an objective assessment and precise structural and functional evaluation of the glottis, and it is crucial for diagnosing various glottic pathologies [10]. Initially used for functional voice disorders, HSV is now increasingly applied to organic glottic lesions [24,25]. Unlike laryngovideostroboscopy (LVS), HSV captures real vocal fold vibrations at thousands of frames per second, enabling the analysis of vibration patterns in asynchronous voices and severe pathologies where strobe synchronization fails [2,5].

This study aimed to assess the effectiveness of generating kymograms for quantifying vocal fold oscillations using LVS and HSV recordings. A total of 247 subjects underwent both stroboscopy and HSV as part of standard diagnostic protocols, providing data for analysis. To our knowledge, this is the largest study assessing glottic pathologies in both methods. Of these, 179 presented with hypertrophic glottic masses, including 123 benign and 56 malignant lesions. The findings confirmed HSV as a valuable method for assessing organic glottic lesions, providing a higher rate of successful kymogram generation compared to LVS, which may aid in the evaluation of vocal fold oscillations. Consistent with previous studies, HSV outperformed LVS in generating kymograms, delivering faster, higher-quality results that address LVS’s inherent limitations [13,26]. HSV-based kymograms allow for more objective evaluations of phonatory vibrations and quantitative parameter analysis, aiding less experienced clinicians in the preoperative diagnosis of glottic hypertrophic masses.

The existing literature highlights the subjective nature of vocal fold vibration assessments in both LVS and HSV examinations that are often influenced by clinician experience. Fujiki et al. explored subjective laryngeal ratings from LVS and HSV by categorizing raters by experience levels (over or under five years) [11]. They found high inter-rater reliability for amplitude, mucosal wave, and non-vibrating portions but noted challenges in assessing phase symmetry and periodicity [11]. In 9% of LVS and 6% of HSV exams, clinicians failed to identify at least one parameter. Similarly, Poburka et al. noted that inter-judge reliability ranged from 0.57 to 0.96 for LVS and from 0.81 to 0.94 for HSV in their assessment of mucosal wave and non-vibrating portions [8]. Efforts to improve objectivity in LVS and HSV assessments, such as through visuoperceptual variables, have been explored [27].

While previous studies have compared LVS and HSV, our research uniquely focuses on the specific challenges and success rates in generating analyzable kymograms across different patient groups, including normophonic individuals and those with functional, benign, and malignant vocal fold lesions. Additionally, our study provides a detailed analysis of the reasons behind LVS failure and the extent to which HSV can overcome these challenges [5,8,9].

For years, our department has used kymographic analysis to evaluate glottal phonatory function, transitioning from LVS- to HSV-based methods [23,28]. Advanced HSV techniques provide objective metrics for vocal fold amplitude, symmetry, and periodicity. Consistent with literature data, HSV-generated kymograms are faster and easier to produce than those derived from LVS [5,29].

Research demonstrates that high-speed videoendoscopy (HSV) significantly outperformed low-speed videoendoscopy (LVS) in generating kymograms. The observed better outcomes for unilateral lesions may be attributed to greater asynchrony in vocal fold vibrations, which HSV captures effectively through kymographic analysis.

In the functional dysphonia group, HSV also showed a significantly higher success rate than LVS; however, no significant difference was observed in the normophonic control group. Nevertheless, in clinical practice, obtaining high-quality recordings in patients with voice pathologies is prioritized, where HSV seems particularly advantageous [30].

Our results are consistent with prior studies suggesting the utility of HSV in diagnosing organic dysphonia [8,31]. Asymmetric hypertrophic glottal masses can often cause asynchronous vocal fold vibrations, which HSV can precisely capture, facilitating the detailed analysis of affected versus unaffected folds [12,32]. In this research, for benign glottic lesions, HSV achieved a 26% higher kymogram success rate than LVS, and for malignant lesions, this advantage rose to 41%. These differences were highly significant and support HSV’s advantage in morphological insights into vocal fold oscillations compared to stroboscopy [10,21]. Additionally, HSV-based kymograms provided accurate measurements of parameters such as amplitude, glottic dynamics, asymmetry, phase difference, and periodicity of vibrations. Figure 9 and Figure 10 highlight the improved quality of HSV kymograms compared to LVS. Consistent with the findings by Powell et al., HSV’s higher frame rate and resolution facilitated the assessment of laryngeal pliability [24]. Yamauchi et al. further noted HSV’s ability to identify non-vibrating areas in infiltrative malignant lesions, such as invasive carcinoma [32].

Our study compared factors affecting kymogram generation with LVS and HSV. Hidden glottal opening (due to prolonged closing phases) was a notable challenge, affecting 7.3% of HSV recordings versus 17% for LVS (*p* = 0.0009). This difference likely arises from LVS sampling incomplete glottal cycles, which is unlike HSV, which captures full cycles, enabling precise measurements of medial vocal fold edge distances. Previous studies confirm that HSV provides a more accurate representation of laryngeal vibrations than LVS [13,21,24]. The limitations of both methods are detailed in Table 2.

Improved lighting in HSV using laser illumination enhanced kymogram quality by increasing the brightness. According to manufacturer specifications and clinical observations, laser illumination did not significantly raise the endoscope’s temperature; however, the potential for localized heating and its effects on the tongue root and vocal tract tissues should be considered when using high-intensity light sources. In our study, HSV captured full-color images, which improved visibility compared to the specific LVS system used, where images were often darker due to the limitations of the LED strobe lamp. However, it is important to note that some LVS systems with higher-intensity strobe lamps can provide bright and well-illuminated images, potentially mitigating this issue. Although some authors suggest that grayscale is beneficial for HSV recordings, others identify insufficient brightness as a common limitation in color HSV recordings [8,9,11].

Another limitation of LVS is its reliance on light synchronization, particularly in moderate or severe dysphonia cases where an unstable voice signal can disrupt synchronization, producing asynchronous sequences that are challenging to interpret. This limitation affected 8.1% of our LVS recordings and was a significant barrier to kymogram generation compared to HSV (*p* < 0.001 for synchronization issues; *p* = 0.0003 for unstable phonation). Prior studies report a 17–63% failure rate for LVS in similar cases [21,38]. HSV, by contrast, proved superior for assessing glottic malignant lesions and cases of asynchronicity, aligning with recommendations for its use in complex pathologies [24].

Our findings provide several new insights into the feasibility and accuracy of HSV compared to LVS for kymographic analysis. We confirmed previous studies indicating the superior ability of HSV in capturing detailed vibratory patterns and overcoming synchronization limitations inherent to LVS. However, our study extends existing knowledge by offering a comprehensive analysis of failure rates and success factors in a larger patient cohort, particularly in cases of organic glottal lesions.

In contrast to earlier research, our results highlight the specific challenges associated with LVS, such as its dependency on phonation stability and lighting conditions, which have not been thoroughly quantified before; however, some technical aspects, like lightning conditions, should not be generalized across all HSV and LVS systems available on the market today. Our findings align with those by Patel et al. (2008) and Poburka et al. (2017) but provide new evidence regarding the practical implications of these limitations in clinical practice [8,21].

In the future perspective, artificial intelligence (AI), which has shown significant potential in the recognition of both functional and non-functional glottic pathologies, may be further investigated in HSV or LVS to help differentiate laryngeal lesions. Studies have demonstrated that machine learning algorithms, combined with imaging modalities such as narrow-band imaging (NBI) and white light imaging (WLI), can enhance the early detection and classification of glottic lesions, including benign and malignant conditions [39,40]. Additionally, AI models utilizing voice signals, demographics, and structured medical records have been developed to differentiate glottic neoplasms from benign voice disorders, offering a non-invasive diagnostic approach with promising accuracy [41].

Overall, our study supports and expands upon the existing literature, reinforcing the advantages of HSV while providing new data on its applicability across different clinical scenarios. Future research should focus on optimizing HSV protocols to further improve diagnostic accuracy and efficiency in voice disorder assessments.

## 5. Conclusions

This study highlights the comparative effectiveness of high-speed videolaryngoscopy (HSV) and laryngovideostroboscopy (LVS) in generating kymograms and objectively assessing vocal fold function in patients with voice disorders. HSV demonstrated a significantly higher kymogram generation success rate (83% vs. 57% for LVS), particularly in cases of patients with asynchronous vocal fold oscillations associated with both organic and functional pathologies, with statistically significant differences across all patient groups, including those with benign and malignant glottic lesions. By overcoming LVS limitations through high frame rates and real-time imaging, HSV enables detailed assessments even in severe dysphonia or extensive glottic lesions. Its ability to provide precise quantitative parameters such as amplitude, asymmetry, and glottal closure underlines HSV’s potential as a routine diagnostic tool, offering improved diagnostic accuracy and outcomes in specialized laryngeal assessments.

Future research could aim to improve HSV accessibility and develop automated data analysis tools to facilitate its clinical integration. Hybrid approaches combining HSV’s detailed accuracy with the practicality of LVS, as explored in our department’s previous work, may further optimize laryngeal imaging practices. Broader adoption of HSV could significantly enhance the quality and precision of voice disorder diagnostics, ultimately leading to better therapeutic outcomes for patients.

## Figures and Tables

**Table 1:** Characteristics of the study group.

Variables	Benign Lesions	Malignant Lesions	Functional Disorders	Control Group
n	123	56	21	47
median age	57	70	59	50
males/females	35/88	46/10	7/14	13/34

**Table 2:** Comparison of limitations of the HSV and LVS techniques based on our experience and the literature cited in the discussion.

**TECHNICAL AND EQUIPMENT LIMITATIONS**	
**HSV**	- expensive equipment limits accessibility. - large datasets require specialized software. - optics can discomfort patients and restrict anatomical visualization.
**LVS**	- limited resolution and brightness reduce detail visibility. (however, the resolution and brightness of LVS recordings in our study were limited by the specific system used, which may not reflect the capabilities of newer high-resolution LVS systems available on the market.) - sometimes, images fail to reflect irregular vibrations, preventing the examination.
**COMPARISON**	HSV provides high-quality, real-time vibratory tracking but entails higher costs and demanding data management. LVS is more accessible and uses flexible scopes but lacks resolution and precision for detailed assessments.
**LIGHTING AND IMAGING SYNCHRONIZATION**	
**HSV**	- HSV requires specialized lighting for optimal imaging, though newer systems are improving illumination technology.
**LVS**	- LVS requires stable phonation for strobe synchronization, as it struggles with strobe synchronization in cases of asynchronic voices or severe dysphonia, reducing interpretable images.
**COMPARISON**	HSV delivers superior imaging, aided by advanced lighting, while LVS often inhibits the examination in severe dysphonic patients lacking stable vocal patterns.
**DEPENDENCE ON STABLE PHONATION**	
**HSV**	- our HSV examination was limited to rigid optics; however, various studies have demonstrated the feasibility of flexible high-speed videoendoscopy [23,31,33,34,35,36,37].
**LVS**	- LVS needs around 10 s of stable phonation, which poses a challenge for patients with severe voice disorders.
**COMPARISON**	LVS relies on stable phonation, which is necessary for light synchronization, limiting its use in severe dysphonia, while HSV effectively captures all irregular vibrations in real time without requiring synchronization.
**STRUCTURAL AND ANATOMICAL CHALLENGES**	
**HSV**	- challenges in visualizing the glottis with anatomical variations (e.g., prominent epiglottis) which disturbs kymogram generation; this is related to the use of rigid optics in our HSV system. - while HSV provides high-resolution imaging, large lesions that cover the glottis may still prevent a full assessment.
**LVS**	- lower resolution and frame averaging hinder the assessment of intricate lesions or non-vibrating areas. - flexible scopes improve comfort but struggle with large masses and reduce resolution in complex cases.
**COMPARISON**	Our HSV system offers clearer imaging but is restricted by rigid scopes, while LVS provides less detail but gains flexibility with adaptable scopes.
**DATA INTERPRETATION AND OBJECTIVITY**	
**HSV**	- HSV result interpretation from the software requires expertise.
**LVS**	- LVS assessments rely on subjective interpretation, causing inconsistencies, especially for phase symmetry and periodicity. - Quantitative analysis in LVS is time-consuming, delaying real-time diagnostic feedback.
**COMPARISON**	HSV offers greater accuracy but requires costly equipment and advanced expertise. LVS is prone to subjective interpretation and observer variability, especially for less experienced users, and objective analysis is time-consuming.

## Data Availability

The data presented in this study are available upon request from the corresponding author due to restrictions regarding patients’ privacy.
